# International society of sports nutrition position stand: essential amino acid supplementation on skeletal muscle and Performance

**DOI:** 10.1080/15502783.2023.2263409

**Published:** 2023-10-06

**Authors:** Arny A. Ferrando, Robert R. Wolfe, Katie R. Hirsch, David D. Church, Shiloah A. Kviatkovsky, Michael D. Roberts, Jeffrey R. Stout, Drew E. Gonzalez, Ryan J. Sowinski, Richard B. Kreider, Chad M. Kerksick, Nicholas A. Burd, Stefan M. Pasiakos, Michael J. Ormsbee, Shawn M. Arent, Paul J. Arciero, Bill I. Campbell, Trisha A. VanDusseldorp, Ralf Jager, Darryn S. Willoughby, Douglas S. Kalman, Jose Antonio

**Affiliations:** a University of Arkansas for Medical Sciences, Center for Translational Research in Aging and Longevity, Department of Geriatrics, Little Rock, AR, USA; b University of South Carolina, Department of Exercise Science, Arnold School of Public Health, Columbia, SC, USA; c Auburn University, School of Kinesiology, Auburn, AL, USA; d University of Central Florida, School of Kinesiology and Rehabilitation Sciences, Orlando, FL, USA; e Texas A&M University, Exercise & Sport Nutrition Lab, Department of Kinesiology and Sports Management, College Station, TX, USA; f Lindenwood University, Exercise and Performance Nutrition Laboratory, College of Science, Technology, and Health, St Charles, MO, USA; g University of Illinois Urbana-Champaign, Department of Kinesiology and Community Health, Urbana, IL, USA; h National Institutes of Health, Office of Dietary Supplements, Bethesda, MD, USA; i Florida State University, Institute of Sports Sciences and Medicine, Nutrition and Integrative Physiology, Tallahassee, FL, USA; j University of Pittsburgh, Department of Sports Medicine and Nutrition, Pittsburgh, PA, USA; k Skidmore College, Health and Physiological Sciences, Saratoga Springs, NY, USA; l University of South Florida, Performance & Physique Enhancement Laboratory, Tampa, FL, USA; m Bonafede Health, LLC, JDS Therapeutics, Harrison, NY, USA; n Jacksonville University, Department of Health and Exercise Sciences, Jacksonville, FL, USA; o Increnovo LLC, Whitefish Bay, WI, USA; p University of Mary Hardin-Baylor, Human Performance Lab, School of Exercise and Sport Science, Belton, TX, USA; q Nova Southeastern University, Dr. Kiran C Patel College of Osteopathic Medicine, Department of Nutrition, Davie, FL, USA; r Nova Southeastern University, Department of Health and Human Performance, Davie, FL, USA

**Keywords:** Protein, exercise

## Abstract

Position Statement: The International Society of Sports Nutrition (ISSN) presents this position based on a critical examination of literature surrounding the effects of essential amino acid (EAA) supplementation on skeletal muscle maintenance and performance. This position stand is intended to provide a scientific foundation to athletes, dietitians, trainers, and other practitioners as to the benefits of supplemental EAA in both healthy and resistant (aging/clinical) populations. EAAs are crucial components of protein intake in humans, as the body cannot synthesize them. The daily recommended intake (DRI) for protein was established to prevent deficiencies due to inadequate EAA consumption. The following conclusions represent the official position of the Society: 1. Initial studies on EAAs’ effects on skeletal muscle highlight their primary role in stimulating muscle protein synthesis (MPS) and turnover. Protein turnover is critical for replacing degraded or damaged muscle proteins, laying the metabolic foundation for enhanced functional performance. Consequently, research has shifted to examine the effects of EAA supplementation – with and without the benefits of exercise – on skeletal muscle maintenance and performance. 2. Supplementation with free-form EAAs leads to a quick rise in peripheral EAA concentrations, which in turn stimulates MPS. 3. The safe upper limit of EAA intake (amount), without inborn metabolic disease, can easily accommodate additional supplementation. 4. At rest, stimulation of MPS occurs at relatively small dosages (1.5–3.0 g) and seems to plateau at around 15–18 g. 5. The MPS stimulation by EAAs does not require non-essential amino acids. 6. Free-form EAA ingestion stimulates MPS more than an equivalent amount of intact protein. 7. Repeated EAA-induced MPS stimulation throughout the day does not diminish the anabolic effect of meal intake. 8. Although direct comparisons of various formulas have yet to be investigated, aging requires a greater proportion of leucine to overcome the reduced muscle sensitivity known as “anabolic resistance.” 9. Without exercise, EAA supplementation can enhance functional outcomes in anabolic-resistant populations. 10. EAA requirements rise in the face of caloric deficits. During caloric deficit, it’s essential to meet whole-body EAA requirements to preserve anabolic sensitivity in skeletal muscle.

## 1. Methods

ISSN position stands are invited papers the ISSN editors and Research Council identify as topics of interest to our readers that need position stands to provide guidance to readers and the profession. Editors and/or the Research Council identify a lead author or team of authors to perform a comprehensive literature review. The draft is then sent to leading scholars for review and comment. The paper is then revised as a consensus statement and reviewed and approved by the Research Council and Editors as the official position of the ISSN.

## 2. Introduction

The dietary “essential” amino acids (EAAs) – histidine, isoleucine, leucine, lysine, methionine, phenylalanine, threonine, tryptophan, and valine – are called “essential” because they cannot be produced endogenously, and thus must be consumed for human survival. In addition, arginine is considered a “conditionally” essential amino acid, meaning that in certain circumstances, the endogenous production of arginine fails to meet physiological needs. The necessity of consuming all the EAAs has been well established over the last 100 years [1], and there are accepted daily requirements for each EAA as part of normal dietary intake [2]. Daily requirements are based on the minimum amount of each EAA that must be consumed to avoid clinical symptoms of deficiency. Inadequate consumption of just one of the nine EAAs will cause symptoms of deficiency, including impaired protein synthesis [3]. Requirements for daily consumption of each EAA are conventionally met as components of routine dietary protein intake. The amount and profile of EAAs in individual dietary proteins, along with the digestibility of the protein bound EAAs, form the basis for the quantitative assessment of the quality of the pure protein [4]. Proteins that contain an abundant amount of all the EAAs in a highly digestible format are considered to be “high-quality” proteins [4].

While the importance of meeting minimal requirements for each EAA through consumption of high-quality dietary protein intake has been recognized for many decades [5], the benefits attainable from consumption of free-form EAAs in amounts above and beyond minimal requirements has only become fully appreciated in the past 25 years. Products of single free amino acids, such as leucine or lysine, and compositions of small groups of EAAs, most notably the branched chain amino acids (leucine, valine, and isoleucine; BCAAs) are available, but many studies have documented that greater benefits are attained from compositions containing all the EAAs. Daily supplementation with compositions of all the EAAs in free form has been shown to be beneficial in many ways [6]. Most prominently, supplemental free-form EAA compositions stimulate protein synthesis and protein turnover throughout the body, including the synthesis of new muscle protein. Stimulation of muscle protein synthesis (MPS) by EAAs can produce gains in muscle mass and quality, which translate to improvements in physical performance and functional outcomes [7].

This document presents the position of the International Society of Sports Nutrition (ISSN) on the effect of dietary supplementation with free-form EAAs on MPS, muscle mass and quality, and physical performance. Evaluation of the benefits of EAAs differs from many of the other nutritional supplements evaluated by the ISSN in that there are well-accepted requirements for daily EAA consumption. Also, rather than supplements containing only a single compound, such as creatine, there are almost limitless combinations of the nine EAAs that can be made depending on the physiological demand. This document will focus on supplementation of the diet in individuals meeting their daily requirements for EAAs through dietary consumption of protein. Only compositions containing the nine EAAs in free form will be considered in this document.

## 3. Mechanism of action

### 3.1. The importance of muscle protein turnover

The continuous renewal of degraded and damaged muscle protein is important in maintaining muscle protein mass and function. In the post-absorptive state, a net breakdown of muscle protein maintains a constant supply of plasma EAAs that provides precursors for protein synthesis in other tissues and organs. EAAs from dietary intake restore the net loss of muscle protein by stimulating MPS. In normal conditions, rates of MPS and muscle protein breakdown are equal over the course of the day. If MPS exceeds the rate of muscle protein breakdown, muscle mass will increase over time, with a potential gain in strength. Accelerated muscle protein turnover (i.e. protein synthesis and breakdown increasing equally) without a gain in net muscle protein mass may also benefit muscle function by replacing older, damaged muscle fibers with new, highly functioning fibers [8]. Thus, stimulation of MPS/turnover is the principal metabolic basis for increasing strength and physical function. While changes in protein breakdown also play a role in controlling muscle protein metabolism, stimulated MPS is the primary basis for the beneficial effects of EAA supplements. Further, early investigations demonstrated that the amino acid effect on skeletal muscle was primarily through the stimulation of MPS, as muscle protein breakdown was unchanged in an acute study [9]. Importantly, since the measurement of muscle protein breakdown is not straightforward and problematic with exogenous intake, MPS also represents a surrogate indicator of protein turnover.

### 3.2. Control of muscle protein synthesis

The most definitive way in which to assess the effect of EAA supplements on physical performance is to measure metabolic and functional outcome responses over time when different amounts and profiles of EAAs are provided, granted that all other variables are held constant. However, months of treatment may be necessary to obtain reliable results due to the slow turnover rate of muscle protein and difficulty in controlling all other variables (diet, total EAA intake, activity, etc.). As a result, use of stable isotope tracer methodology to quantify the acute response of MPS to a single dose of EAAs in human subjects has become the accepted surrogate for predicting the anabolic response in muscle. Aspects of protein synthesis, transcription and translation, can potentially be affected by the consumption of EAAs: translational initiation and elongation in particular (see Figure 1a). The transcription of messenger RNA (mRNA) from DNA results from activation of the relevant genes. Changes in the activation of genes are reflected in the number of specific mRNAs in the cell. The expression of mRNA is important because the physical assembly of new proteins occurs on the mRNA. The complex initiation process consists of several linked stages that are mediated by eukaryotic initiation factors (eIFs). The mammalian target of rapamycin complex 1 (mTORC1) is a key regulator of the activation of downstream eIFs that are mediators of MPS initiation (see Figure 1b). Both transcription and translational initiation of the protein synthetic process can be stimulated by amino acids and exercise [13–16]. However, both mRNA transcription [17] and mTORC1 phosphorylation state [18] are usually poorly correlated with rates of MPS, meaning that neither process is likely to be rate-limiting for MPS in most circumstances. The translational control of protein synthesis by EAA availability has been recognized since 1958 [19]. Translation involves the successive linking of amino acids in the order dictated by the mRNA code. Free intracellular amino acids are bound to corresponding transfer RNAs (tRNAs), forming charged tRNA molecules. Charged tRNA molecules in turn sequentially transfer the attached amino acids to the sites on the mRNA that correspond to the code of the charged tRNA. Translational elongation can only proceed to completion if adequate amounts of all required amino acid precursors are available. A relative deficiency of any EAA will make that EAA limiting, with translational elongation being terminated before the process is complete. Translational control of MPS requires that adequate amounts of all EAAs are available to sustain increased MPS rates. In addition to providing the necessary precursors for protein synthesis, EAAs increase the genes associated with amino acid sensing, transport and mTORC1 regulation [20].

**Figure 1a.**
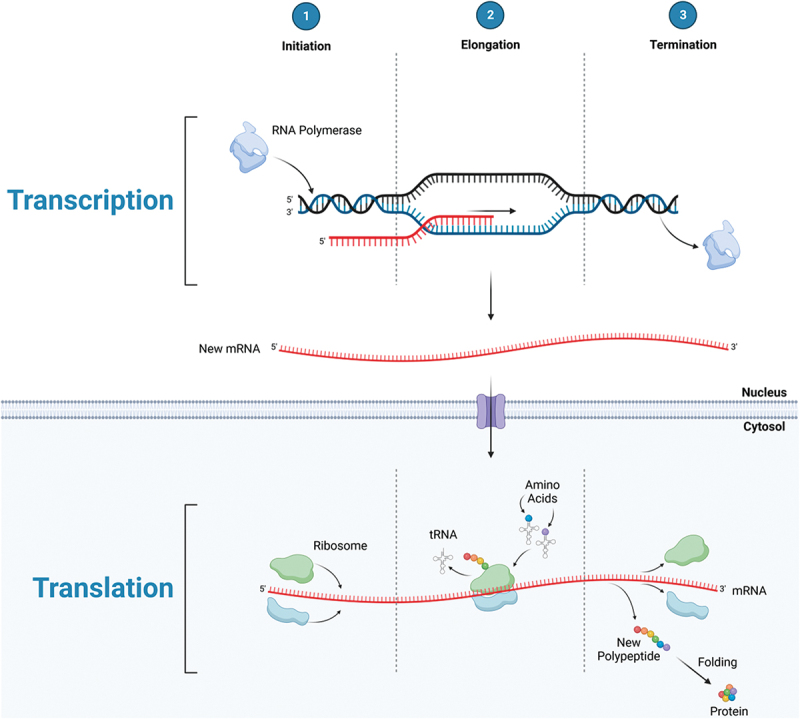
Protein synthesis: transcriptional initiation, elongation, and termination leading to the production of mRNA in the nucleus, then exported to the cytosol to undergo translational initiation, elongation, and termination; producing a polypeptide which is folded into a protein.

**Figure 1b.**
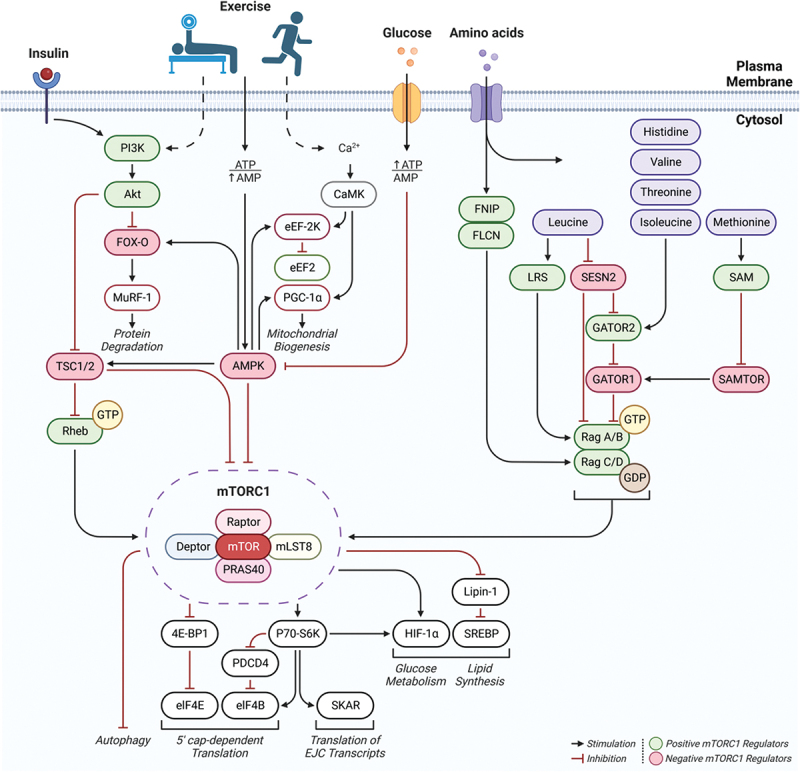
mTOR signaling: regulation of mTORC1 by upstream stimuli; insulin, exercise (resistance, endurance), glucose, and amino acids (Aa’s). Exercise leads to an energy deficit (increased AMP) stimulating AMPK, inhibiting mTORC1 whereas glucose consumption raises ATP, inhibiting AMPK. Insulin and resistance exercise activate the PI3K pathway, positively regulating mTORC1, while endurance exercise activates CaMK, mostly promoting mitochondrial biogenesis. AA’s primarily stimulate mTORC1 by promoting the phosphorylation and dephosphorylation of rag GTPases, rag A/B and rag C/D, respectively. AA’s generally stimulate FNIP1/FLCN, promoting dephosphorylation of rag C/D, however, some EAA’s (leucine, histidine, valine, threonine, isoleucine, methionine) act to promote phosphorylation of rag A/B; leading to an upregulation in mTORC1 and downstream translation as well as glucose and lipid metabolism. Figures are derived from [10–12].

*Abbreviations*: Akt, protein kinase B; AMPK, AMP-activated protein kinase; PI3K, phosphoinositide 3-kinase; Ca^2 +^, calcium ion; CaMK, calcium/calmodulin-dependent protein kinase; FNIP1, folliculin-interacting protein 1; FLCN, folliculin (FLCN); mTORC1, mammalian target of rapamycin complex 1; FOX-O, forkhead box-O transcription factors; PGC-1α, peroxisome proliferator-activated gamma coactivator-1 alpha; MuRF-1, muscle ring-finger protein-1; eEF-2K, eukaryotic elongation factor-2 kinase; eEF2, eukaryotic elongation factor-2; TSC1/2, Tuberous sclerosis proteins 1 (hamartin) +2 (tuberin); Rheb, Ras homolog enriched in brain; LRS, leucyl-tRNA synthetase; SESN2, Sestrin-2; GATOR1/2, GAP (GTPase-activating protein) activity toward Rags 1+2; SAM, s-adenosyl methionine; SAMTOR, s-adenosyl methionine sensor for mTORC1; 4E-BP1, eukaryotic translation initiation factor 4E-binding protein 1; P70-S6K, (S6K1) ribosomal protein S6 kinase beta-1; SREBP, sterol regulatory element binding protein; HIF-1α, hypoxia-inducible factor-1 alpha; PDCD4, Programmed cell death protein 4; SKAR, S6K1 Aly/REF-like substrate; eIF4E/B, eukaryotic translation initiation factor 4E+B.

### 3.3. Importance of the protocol used to measure MPS response to amino acid consumption

The most commonly used method to measure MPS in human subjects is to determine the rate of incorporation of a stable isotope tracer amino acid into muscle protein over time, divided by the precursor enrichment [21]. This approach results in the calculation of muscle protein fractional synthetic rate (FSR). Since muscle mass is relatively constant across several days, changes in FSR are conventionally considered to be a direct reflection of MPS [21]. An alternative approach to measuring MPS is based on the arterial-venous difference of unlabeled and labeled amino acid tracer and tracee, and the isotopic enrichment of the intracellular free pool [22]. These two methods give comparable results for MPS in human subjects [23]. In fact, the acute stimulation of MPS by consumption of EAAs has shown to be reflected in the 24-hour protein balance across the leg [24]. Evidence supports the translation of differences in the acute response of MPS to EAA consumption to outcome parameters measured over weeks or months. For example, the effect of daily consumption of an EAA-based formula in normal young healthy subjects were compared to a placebo over 28 days of complete bed rest [7]. Daily consumption of the EAA-based formula throughout the 28 days of bed rest ameliorated the loss of muscle mass observed in those consuming the placebo by an amount predicted by the pre-bed rest tracer study [7]. The predictive accuracy of the acute tracer method in this paradigm is particularly impactful because activity and dietary intake were completely controlled over the 28-day intervention [7].

The time period over which MPS is determined is important when interpreting the physiological significance of acute changes in response to amino acid intake. A transient increase in MPS is less likely to predict long-term gains in muscle mass and function compared to a response that remains above the baseline value for three hours or more. For example, consumption of leucine alone may elicit a transient response (1–2 h) in muscle MPS, but this response must be interpreted with caution [25]. Consumption of a sufficient amount of leucine alone may activate mTORC1 and associated molecules involved in the initiation of the protein synthetic process and is reflected by a transient increase in muscle protein MPS. However, the synthesis of muscle protein requires adequate availability of all the component amino acids, including all nine EAAs. In the absence of dietary intake, the EAAs needed to produce complete muscle proteins must come from endogenous sources. Initially, the additional EAAs needed for the synthesis of complete muscle proteins may come from pools of free EAAs in the intracellular and extracellular fluid. However, the resulting depletion of free EAAs in those pools will limit muscle protein synthesis because of inadequate precursor (available EAAs in amino acid pools) availability. The only other potential source of the necessary EAAs to maintain MPS in this circumstance is accelerated protein breakdown, which will limit any net gain in muscle protein that might be expected based on acute changes in MPS. Thus, the anabolic response (i.e. MPS – MPB) of muscle protein to consumption of a single EAA, such as leucine, or small groups of EAAs (BCAAs) will be limited by the low availability of the other EAAs.

The paper by Fuchs and associates [26] provides evidence as to the interpretation of the importance of sampling interval on muscle FSR response to amino acid intake. In this study, muscle FSR was determined in response to consumption of the BCAAs, milk protein, or branched chain keto acids, corresponding to leucine, valine, and isoleucine. Muscle protein FSR was stimulated over the first two hours after consumption of all three dietary supplements. However, 2–5 h after ingestion of each supplement, muscle protein FSR remained stimulated only after consumption of milk protein. In other words, ingestion of either the BCAAs or their associated keto acids, failed to stimulate muscle protein FSR from baseline after consumption [26]. The rate of muscle protein FSR was limited in hours 2–5 after their consumption by a decrease in the availability of the EAAs not provided in the dietary supplement, as reflected by the decrease in plasma phenylalanine concentration. The decrease in plasma EAAs was likely ameliorated to some extent by an increased rate of muscle protein breakdown, thereby limiting the net anabolic effect of the stimulation of muscle protein FSR. In contrast, phenylalanine (as a reflection of EAAs) availability was elevated 2–5 h after consumption of milk protein because of continued digestion and absorption of all the EAAs, as well as availability of supporting non-essential amino acids. The authors concluded that “*these data suggest that in addition to the postprandial rise in plasma BCAA concentrations, other (essential) amino acids need to be provided to allow for a more prolonged postprandial increase in muscle protein synthesis rate*” [26]. It is therefore reasonable to rely primarily on data from studies in which MPS has been determined over an interval of 3 h or more after EAA consumption to expect translation of results to functional outcomes.

## 4. Safety

Consumption of EAAs has not been reported to cause adverse responses. Individuals with rare genetic diseases involving impairment of the ability to metabolize certain EAAs, such as maple syrup urine disease (inability to metabolize the BCAAs), could have an adverse response to supplements containing all the EAAs. However, inborn errors of metabolism affecting the metabolism of an EAA are evident at an early age, and dietary adaptation is necessary for health and possibly survival. It is therefore unlikely that an adult with an inborn error of metabolism that limits the safe consumption of EAAs would be unaware of such a condition. It is also possible that an individual with renal disease could react poorly to EAA supplementation, as a low protein diet is often recommended in kidney disease due to the accumulation of urea and ammonia in blood. However, an EAA-based supplement generally does not contribute to increased urea or ammonia production because of increased reutilization of non-essential amino acids for protein synthesis. However, there is insufficient data available on individuals with impaired kidney function to determine the safety of EAA-based dietary supplements.

Limited data are available upon which to base the safe upper limit of individual EAA consumption. Table 1 lists consumption levels of each EAA that has been shown to be safe. The safe upper limits in Table 1 are expressed as the amount of each EAA consumed above habitual intake. Thus, when considering the safe amounts of each EAA, these data indicate that more than 100 g of *supplemental* EAAs can be safely consumed per day in an American adult already consuming the average habitual dietary intake of approximately 40 grams per day. Reasonable dosage of an EAA supplement does not exceed 15 g, meaning that even as much as three maximal doses per day is in line with normal daily consumption of EAAs through dietary protein food sources. The following data on the effects of supplemental EAA were derived in populations with adequate dietary protein intake, unless stated otherwise.

**Table 1.** Dietary requirements of essential amino acids and safe upper limit of consumption for adults.

Amino Acid	Requirement (mg.kg/day)^1^	Safe upper limit mg/kg/day^2^ (reference)
Histidine	18	64 [27]
Isoleucine	23	117 [28]
Leucine	49	657 [29]
Lysine	48	71 [30]
Methionine	23	71 [31]
Phenylalanine	48	143 [32]
Threonine	28	85 [33]
Tryptophan	8	64 [34]
Valine	32	97 [2]

^1^Refs [2,4], based upon DRIs; ^2^Above habitual intake.

Key points:

- Protein turnover ensures the continuous renewal of degraded and damaged muscle protein and is important in maintaining muscle protein mass and function.
- The accepted surrogate for the measurement of protein turnover is the determination of muscle protein synthesis by stable isotope tracer methodology. Though protein breakdown is important in this process, the primary acute response of EAA intake on skeletal muscle is the stimulation of protein synthesis.
- The safe upper limit of daily EAA intake permits substantial supplementation.

## 5. Consensus of FIndings

### 5.1. Eaas and muscle protein synthesis at rest

MPS is stimulated by consumption of EAA compositions [35] and inhibited by a reduced availability of plasma EAAs [36]. The magnitude of increase in MPS following EAA consumption is a function of the amount ingested. At rest, an oral EAA dose as small as 1.5 g has been reported to stimulate MPS [37] while the maximal effective dose, after which no further stimulation of synthesis is achieved in a single dose, is thought to be 15–18 grams of EAA [38]. The stimulation of MPS by EAA consumption does not require simultaneous consumption of non-essential amino acids (NEAAs) [35,38]. Inclusion of NEAAs to a mixture of 18 g of EAAs in the profile of beef protein had no effect on the EAA-mediated stimulation of MPS [35]. Whereas consumption of NEAAs has no effect on MPS when less than 18 g of EAAs are consumed, it is possible that when more than 18 g of EAAs are consumed NEAAs become limited by maximal rates of endogenous production; however, more research is needed to explore this possibility.

EAA supplements stimulate MPS more than an equal amount of high-quality protein either as an isolate [39] or as a component of a meal [40]. A oral 3 g dose of EAAs was found to stimulate MPS to a similar degree as 20 g of whey protein isolate, which contains approximately 10 g of EAA [41]. Further, the addition of EAAs to whey protein has been shown to significantly enhance the MPS response beyond whey protein alone [42]. The superior stimulatory effect of free-form (individual EAAs only) EAAs is related to the greater amount of EAA/gram compared to a dietary protein source [43]. Due to the high rate of intestinal absorption of free-form EAAs [44], rapid increases in circulating plasma EAA concentrations drive inward transport into the muscle [23,45] resulting in a faster achievement of peak intramuscular EAA concentrations compared to other dietary protein sources. The importance of plasma EAA concentrations and the speed of increase to the peak concentration on the MPS response is unclear, but some studies have found a relation between plasma EAA concentrations and MPS [46] and analysis of consolidated data shows a correlation between the speed of rise to peak concentrations of EAAs and MPS [43]. On the other hand, there was no difference in the MPS response when the same dose of EAAs was given either as a single bolus or five smaller doses over time [47]. Thus, there is agreement of a relationship between the dose of EAAs and the MPS response, but the regulatory mechanisms linking dose and MPS are not agreed upon.

**Figure 2.**
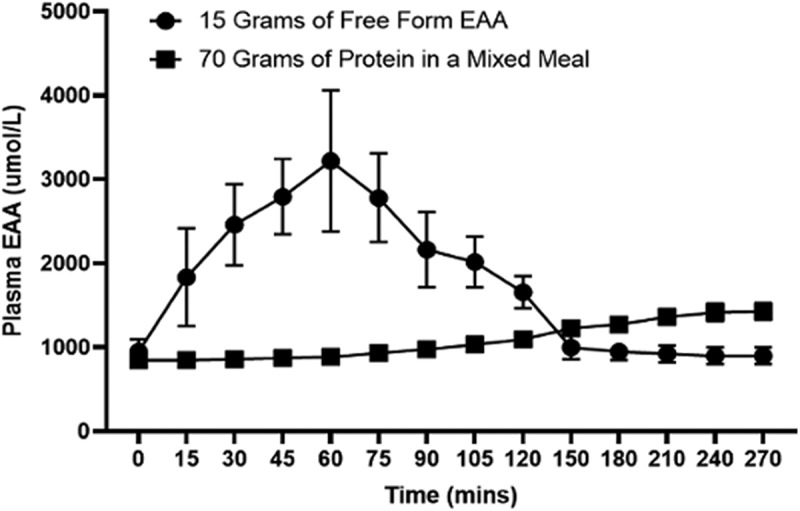
The effect of 15 g of free-form EAA vs 70 g of lean beef protein and mixed meal ingestion on plasma EAA kinetics. Adapted from references [50,51].

The overall impact of a dietary supplement on MPS over 24 hours depends not only on the acute response to the consumption of the composition, but also on the anabolic responses to normal meals. It is well established that the anabolic response to a meal is reduced following pre-loading with a protein isolate [48]. In contrast, a 15 g dose of EAAs had no impact on the anabolic response to the subsequent meal [49]. However, it is important to note that free-form EAA supplementation results in a much greater rise in blood EAA concentrations than a meal of greater EAA quantity, as they require no digestion and are quickly absorded. Figure 2 demonstrates the effects of 15 g of free-form EAA [50] on plasma concentrations compared to a mixed meal containing 70 grams of protein from beef protein [51]. Due to the slow digestion and release of dietary EAA after mixed meal consumption, blood levels are increased only minimally, as compared to a rapid and robust increase following crystalline/free-form EAA ingestion. Moreover, this rapid rise (a 3-fold or greater increase) predicts a greater response of MPS [43].

The physiological state can affect the MPS response to EAAs. Aging is the most commonly studied non-clinical state in which the response to EAAs may be altered, termed anabolic resistance. Decreased responsiveness of MPS to consumption has been well documented [52,53], but not consistently observed [54]. The different MPS responses of older individuals to EAA consumption may be explained by differences in the EAA profile of the composition. For example, on one occasion Katsanos et al. provided elderly subjects a mixture of 6.7 g of EAAs in the profile found in whey protein (27% leucine), and on a second occasion provided the same amount of a mixture of EAAs with leucine comprising approximately 40% of the total of EAAs [55]. In this study, the 40% leucine composition stimulated muscle protein synthesis approximately 50% more than the lower leucine profile, despite containing the same amount of total EAAs [55]. These results demonstrate the potential importance of the EAA profile in a composition. However, there are almost limitless combinations of the nine EAAs, and there is minimal data directly comparing the effectiveness of different EAA profiles in the same circumstance. Due to a lack of sufficient comparative data, the impact of different EAA profiles on MPS will not be discussed in this document. Further research is required in this regard to determine optimal profiles for various physiological requirements.

### 5.2. Eaas and whole-body protein and energy balance

A net negative balance in proteins throughout the body other than muscle protein (i.e. tissues and organs; reflected by whole-body rates of protein synthesis and breakdown) will adversely affect muscle protein and thus physical performance. If an inadequate amount of EAA precursors are available from dietary intake to meet the demand throughout the body, the breakdown of muscle protein and release of amino acids into the blood will provide the necessary EAAs. Negative energy balance will also indirectly affect muscle protein, as ingested EAAs will at least in part be directed to oxidation for energy production rather than being channeled to muscle protein synthesis [56]. A discussion of EAA effects on muscle protein synthesis must therefore be considered in the context of the status of whole-body protein and energy balance.

Periods of caloric deficit are common in weight class and endurance sports such as marathon running and distance swimming, where periods of intense training coupled with the desire for low body weight may limit caloric intake [57]. Caloric deficit increases whole body EAA requirements [58]. For example, five days of a 30% calorie deficit required a 3-fold increase in EAA intake to produce a positive whole-body protein balance [58]. Failure to meet the increased whole-body EAA requirements will result in the net breakdown of muscle protein to provide the necessary EAAs and cannot be fully reversed until the whole-body requirements are satisfied.

Many clinical states induce changes in whole-body protein metabolism that affect EAA requirements and muscle protein balance. There may be new demands for EAA precursors for functions such as repair of damaged tissue, wound healing, and production of acute phase proteins. Coincidently, the normal anabolic response of skeletal muscle to dietary EAAs is likely to be diminished (anabolic resistance) with these conditions. As a result, rapid loss of muscle mass is a common complication of serious diseases and injury [59]. The same response may occur, albeit to a lesser extent, following a strenuous workout or competitive event, particularly in those athletes who are voluntarily or involuntarily in a caloric deficit.

#### 5.2.1. Key points: EAA effects on muscle and whole-body protein

- There is a dose-response of oral EAA on skeletal muscle protein synthesis which plateaus at approximately 15–18 g.
- There is a relationship between plasma EAA kinetics and the stimulation of protein synthesis.
- Oral EAA stimulate muscle protein synthesis to a greater extent than an equal amount of high-quality protein.
- The decreased anabolic response with aging requires a different EAA profile, most notably a greater proportion of leucine.
- Whole-body EAA requirements increase with caloric deficit. If this requirement is not met, net breakdown of muscle protein will occur to furnish the necessary EAAs.

### 5.3. EAAs and physical function in absence of exercise training

Several studies document that acute stimulation of MPS by free EAA compositions translates to long-term gains in muscle mass and function, even in the absence of control for dietary protein intake. Outcome studies have generally been performed in older individuals. Using a randomized, double-blinded, placebo-controlled design, older women were assigned to receive either placebo, or 15 g EAA/d for three months [60]. Ingestion of 7.5 g EAA acutely stimulated muscle protein FSR in both groups at baseline [60]. Basal FSR at three months was increased only in those receiving daily EAA supplementation, and the magnitude of the acute response to EAA was unaltered after three months of EAA consumption. Consistent with the muscle protein FSR data, lean body mass was increased significantly in those receiving EAAs but not placebo [36]. In a similar study, 12 glucose-intolerant subjects ingested 11 g of EAAs two times per day between meals for 16 weeks [61]. Diet and physical activity were not otherwise modified. Lean body mass was increased by EAA consumption, and most importantly, a variety of parameters of physical function were also improved [61]. In a group of 38 older women (≥75 years), daily supplementation with 3 g of EAAs twice per day for three months significantly improved walking speed [62]. Similar results were observed in a study including 92 low-function older individuals given either supplements of 15 g whey protein isolate, EAAs (12 g EAAs plus 3 g flavoring), or nutrition education for 12 weeks [63]. Those receiving EAAs significantly improved the distance walked in 6 minutes, grip strength, and leg strength (peak torque measured by Cybex). Those receiving whey protein also significantly improved the distance walked in 6 minutes, but the improvement was significantly less than those receiving EAAs. Leg strength was not improved in the whey group. Interestingly, the distance walked in the nutrition education group declined over the 12-week intervention period [63]. As a testament to the potential positive impact of EAA supplementation on functional improvements, the magnitude of improvement observed in the 6-minute walk distance in those receiving EAAs was approximately the same as reported in a systematic review of studies reporting the result of 2–6 months of resistance training in 241 individuals [64]. These results are consistent with extrapolation of the acute effects of EAA administration on the control of MPS [46] by blood pharmacokinetics [43]. In a study of healthy older individuals confined to bed rest for 10 days, consumption of three doses of 15 g EAAs per day mitigated declines in physical function evident in the subjects receiving the caloric-equivalent placebo [65]. In summary, existing studies on the effects of EAAs on functional outcomes in the absence of exercise training have largely focused on aging or compromised populations. As the EAA are potent stimulators of muscle and whole-body protein anabolism, these populations are logical targets of primary investigation as they manifest an anabolic resistance in skeletal muscle that leads to muscle loss, functional weakness, comorbidities, and otherwise poor clinical outcomes. Thus, it is tempting to recommend additional studies to determine the anabolic effect of EAAs in young healthy individuals in the absence of exercise training.

### 5.4. EAA interaction with exercise

**Figure 3.**
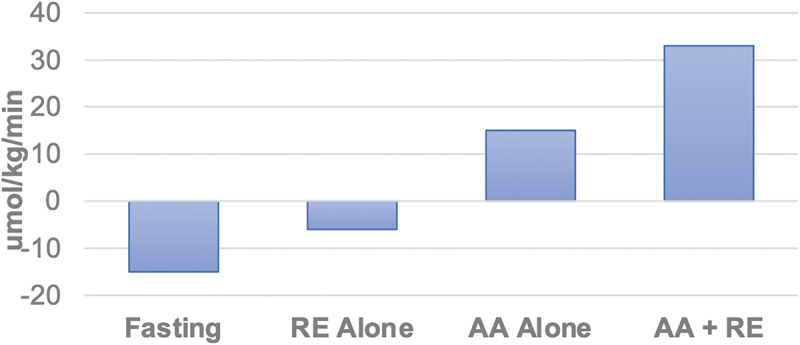
Muscle net balance of phenylalanine (umol/kg/min) during fasting, resistance exercise alone (RE), complete amino acid administration/infusion alone (AA), and with combined AA and RE. An interactive effect between RE and AA administration is demonstrated. Data derived from references [9,66].

Early cross-limb examination of amino acid effects on skeletal muscle demonstrated that increased amino acid delivery at rest results in a stimulation of inward amino acid transport into skeletal muscle (30–100%; dependent upon individual EAAs), a stimulation of protein synthesis (30–300%), and an improvement to a positive net amino acid balance [9]. When lower-body resistance exercise was performed prior to amino acid infusion, greater effects were realized in the inward transport of some amino acids, but more importantly, there were even greater increases in protein synthesis and net muscle protein balance [9]. In each case, there was no change in muscle protein breakdown. It is important to note that the combined effects of resistance exercise and increased amino acid delivery are interactive. When the same resistance exercise was performed without amino acid administration, there was both an increase in inward transport of amino acids as well as muscle protein synthesis; however, net muscle balance remained negative [66]. The absence of improvement in net balance was due to the upregulation of both protein synthesis and breakdown after resistance exercise alone [66]. Thus, resistance exercise alone does not result in muscle anabolism (net muscle protein balance is negative). Anabolism occurs only when supported by the requisite amino acid precursors. Figure 3 graphically represents the cross-limb balance of phenylalanine (a surrogate for amino acid balance since it is not metabolized in skeletal muscle) when fasted, after resistance exercise alone, after amino acids alone are infused, and with combined infused amino acids and resistance exercise. The interactive effects of orally administered EAA and resistance exercise revealed similar results. Lower body resistance exercise was followed by the administration of oral EAA or a complete amino acid mixture. The results indicated that EAA after exercise improved muscle net balance to the same extent as a complete mixture, providing additional evidence [38] that when given in conjunction with exercise, only the EAA are required to stimulate muscle anabolism [67]. Bolus oral ingestion of EAA after lower body resistance exercise also increased protein synthesis and muscle net balance, regardless of whether the drink was consumed one or three hours after exercise [68]. There are indications that the benefits of EAAs on skeletal muscle may not entirely rely on the stimulation of MPS. EAA supplementation improved histological evidence of muscle damage and reduced the loss of muscle strength, even in the absence of any changes in MPS [69].

The interactive effects of EAAs and resistance exercise are reflected in translation initiation signaling in the muscle. Volunteers were given a placebo, leucine, BCAA, or EAA solution after resistance exercise. The results indicated that 90 min following exercise recovery, activation of ribosomal protein S6K1 and eukaryotic translation initiation factor 4E-BP1, as well as a sustained reduction of 4E-BP1:eIF4E interaction, was greatest with EAAs [70]. While a 9-fold increase was observed for S6K1 expression in the EAA trial, the overall stimulation of translation initiation was most effective with EAAs, resulting in progressive increases in translation initiation (placebo < leucine < BCAAs < EAAs) [70]. A study involving young men corroborated the upregulation of the mammalian target of rapamycin complex 1 (mTORC1) signaling pathway after resistance exercise but also noted that an EAA supplement maintains mTORC1 in peripheral regions of muscle fibers that is closer to its direct activator Rheb [71]. The authors hypothesized that the “*intracellular localization of mTOR may serve to prime the kinase for future anabolic stimuli*” [71]. The effects of EAA and resistance exercise upon upregulation of anabolic signaling is consistent with aging. EAAs plus resistance exercise increases peripheral EAA concentrations equally in both young and older subjects [72]. Regardless of age, increases in mTOR (Ser2481) and ribosomal protein S6 (Ser235/236) were demonstrated with the combined treatment [72], suggesting an improved muscle sensitivity to the combined stimuli. further, post-exercise EAA administration in older men leads to an increase in satellite cell proliferation [73]. The metabolic product of this signaling is an improvement in muscle protein synthesis. In addition, older women performed unilateral knee extension and then consumed 1.5 or 6 g of an EAA formula with varying amounts of leucine, or 20 or 40 g of whey protein. The results indicated that 1.5 g and 6 g doses of EAAs were as effective as 40 g of whey protein in the stimulation of acute muscle (myofibrillar) protein synthesis [37].

### 5.5. Supplementation strategies: EAA timing

Timing of EAA administration in relation to resistance exercise must be considered. Subjects given EAAs immediately before or after resistance exercise both realized a 130% increase in arterial and muscle phenylalanine concentrations [74]. However, the effect on net phenylalanine uptake (an indirect measure of net protein synthesis) was much greater when the drink was consumed just prior to exercise [74]. It is important to note that each treatment resulted in a positive net phenylalanine balance; however, the improved amino acid delivery (blood flow X arterial EAA concentration) when consumed immediately before exercise resulted in an approximate 3-fold greater delivery of amino acids [74]. The stimulatory effect of resistance exercise on muscle blood flow, when combined with the greater amino acid delivery from consumption prior to exercise, results in a greater anabolic response in skeletal muscle. This is consistent with a recent review denoting the relationship between peripheral EAA increases and the stimulation of muscle and whole-body protein synthesis [43]. Additional work does not confirm these findings, as a carbohydrate/EAA solution given before resistance exercise did not enhance post-exercise muscle protein synthesis [75]; however, methodological differences/interpretations and carbohydrate intake may complicate the consistency in interpretation. Further, evidence indicates that a second EAA bolus one hour following a first duplicates the muscle anabolism of the first administration, indicating that synthetic mechanisms are not dormant after an initial stimulation [76].

### 5.6. EAA interaction with other exercise modalities

Due to the strong interactive effects demonstrated with EAAs and resistance exercise, the preponderance of work has focused on this combination. The data on the combination of EAAs and aerobic exercise is also consistent with its effects on skeletal muscle protein anabolism. In young adults performing 90 min of intensity matched cycle ergometry or weighted load carriage (30% of body mass) exercise on a treadmill with or without EAAs (consumed every 30 minutes throughout exercise bout), muscle protein synthesis was greater during each exercise mode with EAAs [77]. However, load carriage and EAAs resulted in a greater increase in MPS both during and after exercise [77]. These results indicate that the loading of skeletal muscle provides a stronger stimulus for the combined effects of EAAs and exercise. The interaction of EAAs and aerobic exercise is also consistent in aging. Older volunteers (72 ± 1 yrs) were randomized to receive 15 g/d of EAAs or 15 g/d EAAs plus 3 d/wk supervised aerobic exercise training. Consistent with previous findings, acute EAA intake pre-intervention increased MPS [78]. However, post 24-week intervention, the combined EAAs and aerobic exercise group had a greater muscle synthetic response to EAAs than the EAA group alone [78], indicating that consistent exercise further sensitizes skeletal muscle to the anabolic effects of EAAs. Most importantly, the improved sensitivity of skeletal muscle to EAAs resulted in greater muscle quality and 400 m walking speed in the EAA plus aerobic exercise group [78].

High-intensity and intermittent exercise/training (HIIT), and most team sports (i.e. soccer, basketball, hockey, tennis, etc.) require components of both aerobic and anaerobic/resistance training. Studies with protein suggest that high-intensity exercise undertaken with increased protein availability (i.e. performed in a fed state with protein) may synergistically enhance muscle hypertrophy [79]. Other potential benefits include increased mitochondrial biogenesis, exercise recovery, aerobic capacity, and improved sprint performance [80]. To date, the limited investigations utilizing EAA and HIIT are not yet definitive. In untrained, overweight/obese adults, 8 weeks of high-intensity interval training (HIIT; 6–10 × 1 min@90% max W: 1 min rest) elicited significant increases in thigh muscle size, cross-sectional area, volume, and quality, but was not synergistically enhanced by a low dose of EAA (3.6 g twice daily) [81]. In addition, VO_2_ was also increased with HIIT but not synergistically enhanced by EAA [82]. In the same cohort, there were no acute (3.6 g pre-exercise) or chronic (after 4 and 8 weeks of 3.6 g twice daily) effects of EAA supplementation on time to exhaustion or workload progression [83]. While there is no evidence to suggest EAA supplementation is detrimental or attenuates physiological adaptations associated with HIIT, there is not enough evidence to draw conclusions as to the benefits on adaptation and performance. However, there is reason to expect an EAA interaction with aerobic exercise. Moderate treadmill walking alone (45 min at 40% peak VO_2_) in both young and older men increased muscle protein synthesis immediately after exercise, with the younger response maintained up to one hour post- exercise [84]. Synthesis of fibrinogen was also elevated in both groups up to three hours post-exercise [84]. Amino acid delivery was greater immediately post-exercise, again denoting the exercise effect on limb blood flow. Thus, if increased blood flow is combined with a greater delivery of amino acids by oral intake, one would expect muscle anabolism.

Regarding exercise interactions, free form EAAs may be considered over intact protein (whey) on the basis of ease of consumption proximal to and during exercise. Oral free-form EAA formulas require little to no digestion, entail minimal gastric load, and are rapidly absorbed and transported to the periphery. For this reason, they are ideal for consumption prior to the performance of rigorous exercise.

## 6. Eaas and clinical conditions and outcomes

Beneficial effects of supplementation of the diet with EAAs have been demonstrated in a wide variety of clinical conditions. Conditions largely associated with aging have been a frequent target of EAA therapy, including sarcopenia [62,85], long-term-care-acquired infections [86], low physical function [63], and heart failure [40,87,88]. Beneficial effects of EAAs have also been reported in the following conditions or situations: rehabilitation [89–92]; stroke [93,94]; bed rest/immobilization [8,65,95–97]; peripheral artery disease [98]; renal failure [99–103]; inflammation [104,105]; critical illness [106]; lung cancer [107]; cystic fibrosis [108]; chronic obstructive pulmonary disease [109–111]; wound healing [112]; brain injury [113,114]; metabolic syndrome and cardiovascular risk factors [115–117]; obesity [118,119]; liver fat [115,120–122]; and diabetes [123–127]. Importantly, in all of these studies beneficial effects were observed despite the absence of control of the consumption of EAAs contained in dietary protein, implying the importance of the rapid and complete absorption of free EAAs in clinical circumstances in which digestion may be impaired and anabolic resistance is prevalent. The findings in clinical populations point to the need and potential impact of EAA supplementation surrounding rehabilitation efforts from orthopedic injuries and associated surgery. These scenarios represent the most likely clinical environments where competitive athletes will find themselves and it remains an under-served area of research.

### 6.1. Key points: EAA, exercise, and function

- The anabolic effects of EAA and exercise are interactive, in that the response to the combination is greater than the sum of the individual responses. The increase of exercise-induced limb blood flow is contributory to this response by increasing EAA transport into skeletal muscle.
- The combination of EAAs and aerobic exercise, as well as other modalities of muscle loading, is also anabolic to skeletal muscle.
- EAA given prior to exercise results in greater anabolism than if consumed after completion of exercise; however, a lesser anabolic effect can be realized within an hour after exercise cessation.
- In the absence of exercise stimulus, EAA administration in anabolically resistant populations, such as aging and clinical pathologies, has been shown to be beneficial to clinical outcomes and efficacious in the restoration of strength and functional performance.

## 7. Remaining questions

The metabolic effects of EAAs have been elegantly articulated. In addition, their metabolic interaction with both resistance and aerobic exercise have been established. Thus, there is a metabolic basis for the translation of EAA supplementation to performance outcomes. As mentioned previously, the substantial anabolic effect of free-form EAA supplementation is consistent with their efficacy in populations that would most benefit from their utilization. For this reason, the overwhelming majority of longitudinal work examining EAA supplementation and functional outcomes has occurred in aging or clinical populations characterized by muscle anabolic insensitivity, muscle loss, and/or muscle weakness. Less work has documented the long-term effects/outcomes associated with EAA utilization in athletic populations. In addition, further work defining the optimal profile of EAAs, as well as the optimal dosage and timing for specific circumstances, would be beneficial.

## 8. Final summary and conclusions

The following 15 points constitute the Position Statement of the Society. They have been approved by the Research Council of the Society:

1. Free-form EAA supplementation (not derived from exogenous intact protein) is a robust stimulator of muscle protein synthesis and turnover.
2. EAAs stimulate muscle protein synthesis more than an isonitrogenous protein isolate.
3. EAA ingestion produces a rapid rise in peripheral concentrations and inward transport of amino acids into skeletal muscle.
4. EAA stimulation of muscle protein synthesis can occur with multiple dosages and does not interfere with meal effects.
5. Individual or groups of EAAs may initiate the stimulatory process; however, significant and sustained stimulation occurs when all EAAs are consumed.
6. EAA stimulation of protein synthesis at rest occurs in dosages ranging from 1.5 g to 18 g.
7. A greater percentage of leucine (%/g) contained in ingested compositions of EAAs is required to maximally stimulate muscle protein synthesis populations (aging, clinical pathologies) that demonstrate anabolic resistance.
8. In anabolic resistant populations, longitudinal EAA supplementation improves functional outcomes.
9. The effects of EAAs and exercise are interactive, such that the combined effects are magnified. This interaction is due to a greater delivery of EAAs to exercising muscle by increased blood flow and higher blood EAA concentrations.
10. Anabolic responses are consistently reported with combinations of EAA ingestion with either resistance or aerobic exercise. This effect is preserved with aging.
11. Free form EAA supplementation is well within the safe upper limit of habitual daily consumption.
12. EAA supplementation is efficacious in the vast majority of clinical studies and conditions.
13. Numerous longitudinal studies involving EAA supplementation in aging populations consistently report favorable improvements in metabolic as well as functional outcomes.
14. More research is needed to examine the potential impact of EAA administration in athletic populations that are intentionally or unintentionally undergoing energy deprivation on changes in muscle protein metabolism and associated performance and body composition changes.
15. More research is needed to examine the role of EAA administration to athletic populations that go through unexpected and sudden periods of inactivity likely secondary to acute injuries and rehabilitation periods that routinely follow surgical interventions.
